# Use of Learning Media by Undergraduate Medical Students in Pharmacology: A Prospective Cohort Study

**DOI:** 10.1371/journal.pone.0122624

**Published:** 2015-04-07

**Authors:** Joanna Gutmann, Felizian Kühbeck, Pascal O. Berberat, Martin R. Fischer, Stefan Engelhardt, Antonio Sarikas

**Affiliations:** 1 Institute of Pharmacology and Toxicology, Technische Universität München, Munich, Bavaria, Germany; 2 TUM MeDiCAL Center of Medical Education, Technische Universität München, Munich, Bavaria, Germany; 3 Institut für Didaktik und Ausbildungsforschung in der Medizin, Klinikum der Ludwig-Maximilians-University, Munich, Bavaria, Germany; Penn State College of Medicine, UNITED STATES

## Abstract

The ubiquity of the internet and computer-based technologies has an increasing impact on higher education and the way students access information for learning. Moreover, there is a paucity of information about the quantitative and qualitative use of learning media by the current student generation. In this study we systematically analyzed the use of digital and non-digital learning resources by undergraduate medical students. Daily online surveys and semi-structured interviews were conducted with a cohort of 338 third year medical students enrolled in a general pharmacology course. Our data demonstrate a predominant use of digital over non-digital learning resources (69 ± 7% vs. 31 ± 7%; p < 0.01) by students. Most used media for learning were lecture slides (26.8 ± 3.0%), apps (22.0 ± 3.7%) and personal notes (15.5 ± 2.7%), followed by textbooks (> 300 pages) (10.6 ± 3.3%), internet search (7.9 ± 1.6%) and e-learning cases (7.6 ± 3.0%). When comparing learning media use of teaching vs. pre-exam self-study periods, textbooks were used significantly less during self-study (-55%; p < 0.01), while exam questions (+334%; p < 0.01) and e-learning cases (+176%; p < 0.01) were utilized more. Taken together, our study revealed a high prevalence and acceptance of digital learning resources by undergraduate medical students, in particular mobile applications.

## Introduction

The ubiquity of the internet and multitude of available learning media in higher education has an increasing impact on the way students access information for learning. The current generation of undergraduate students grew up with information and communication technology (ICT) as an integral part of life. This generation of learners born between 1980 and 1994 were termed as “digital natives" [1] or “net generation” [2] due to their presumed familiarity and reliance on ICT. It was suggested that this cohort differs from previous generations of students by their use of technology for knowledge acquisition [1,2]. In addition, some authors proposed differences in learning style and behavior of "digital natives" in comparison to their predecessors. For instance, digital natives were suggested to be experiential learners, proficient in multitasking, comfortable with multimedia learning environments and the use of ICT for interacting with peers and educators [1–4]. This led in part to the assertion that the present educational system might not be fully prepared to deal with the needs and expectations of today`s student generation [5].

This concept has been challenged by a number of educational researchers.

Amongst others, Bennett et al. [6] and Thompson [7] criticized the limited underlying empirical evidence and concluded that the digital natives`approach to learning and technology use varies and is complex rather than deterministic. In addition, there is an ongoing debate surrounding the kind and level of learning media use by today`s students and faculty in higher education [8]. For undergraduate medical education, some studies identified digital media as the predominant information source for medical students [9–11], while other authors reported non-digital resources, notably textbooks, as medium of choice for personal study [12].

A number of studies have investigated the use or acceptance of distinct learning media or technology, e.g. e-books [13], mobile devices [14–17] or Web 2.0 tools [18,19] in higher education. However, only limited data is available on the quantitative and qualitative use of different learning resources by students in a genuine educational context and its longitudinal alterations.

The aim of this study was to systematically analyze the use and acceptance of learning resources by undergraduate medical students. For this purpose we monitored the daily use of digital and non-digital learning media during a teaching module of pharmacology by an online questionnaire, and analyzed the overall media use and media choices in teaching vs. self-study periods. Finally, semi-structured interviews were conducted at the end of the course to gain insights into the learning media preferences by students.

## Methods

### Study design and participants

**Fig 1:**
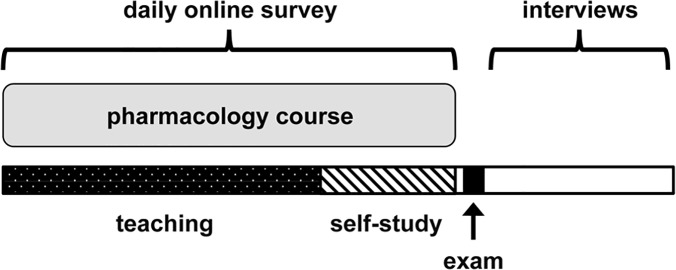
Experimental setting and timeline. A mixed-methods design of quantitative (online surveys) and qualitative (semi-structured interviews) studies was employed during an undergraduate pharmacology course at Technische Universität München, Germany. The course consisted of a 28-day teaching period with daily lectures and twice-weekly seminars, followed by a 10-day self-study period and a final written exam.

A two-phased sequential mixed-methods explanatory design was employed to study the use of learning media by undergraduate medical students. The study was conducted with a cohort of 338 third year medical students enrolled in a general pharmacology course at Technische Universität München (TUM). The module consisted of a 28-day teaching period with daily lectures and twice-weekly seminars, followed by a ten-day self-study period and a final written exam (Fig 1).

Quantitative data were solicited during the lecture and self-study periods by a daily online questionnaire. In addition, a paper-based survey was conducted with all course participants at the end of the self-study period. For descriptive statistics of learning media use, the portions of votes per medium per day were calculated and averaged for the whole study period. For qualitative analysis, semi-structured telephone interviews were conducted after the exam.

The study protocol and consent procedure were approved by the ethics committee of TUM School of Medicine (project number 5701/13). Study participation was voluntary and informed consent was obtained from all study participants via an online form. All data were processed in an anonymized manner.

### Data collection and instruments

To collect quantitative data on learning media use in real time, we developed a web-based survey tool that displayed a questionnaire to study participants in a daily manner. The survey tool was written in Hypertext Preprocessor (PHP) as server-side programming language to be compatible with all major operating systems, and linked to a My Structured Query Language (MySQL) database for storage and analysis of survey data. The user interface consisted of a questionnaire soliciting information on the most, 2nd most and 3rd most used learning medium of the previous day. For each question, study participants could choose one item from a list of ten different learning resources (textbooks > 300 pages, textbooks < 300 pages, lecture slides, software applications for mobile devices (apps), internet search, e-learning cases, podcasts, e-books, personal notes, exam questions), or opt not to respond. The questionnaire was automatically displayed to study participants when visiting the online learning platform (www.tum300.de) used for pharmacology teaching at TUM. A cache memory function prevented multiple daily votings by single users.

### Interviews

Qualitative data was obtained by semi-structured telephone interviews with a sub-cohort of study participants. Following questionnaire data analysis, an interview guide was generated and pilot tested with non-participating students for clarity, relevance and interview length. Convenience sampling was used to recruit interview participants. All interviews were conducted by one researcher (JG) in the weeks following the exam and lasted ten to 15 minutes. After eleven interviews, thematic saturation was reached. The interviews were audio-recorded and transcribed verbatim using f4 transcription software (Dresing & Pehl, Marburg, Germany). Elements of grounded theory and constant comparison were used to identify and develop themes iteratively from ongoing data collection analysis [20,21]. The process involved line-by-line examination of each transcript by the interviewer and coding of phrases into themes.

### Statistics

Graphs are presented as mean ± standard deviation of the mean (SEM). To determine if the two sets of data are significantly different from each other, Student`s t-test was used. A two-way analysis of variance (ANOVA) with Tukey’s multiple comparisons post hoc-test was used to compare the use of digital and non-digital media by male and female students. For statistical analysis, GraphPad PRISM 6.0 (La Jolla, CA) software was used. P values < 0.05 were considered statistically significant.

## Results

### Demographic data

A total of 258 out of 338 (76%) students enrolled in the pharmacology course (winter term 2012/13) at TUM participated in the study. The mean daily participation in the online survey was 79.5 (± 18.2) students (S1 Table). Responses of day 1 and 35 were omitted from further analysis due to low participation numbers (29 and 24 students, respectively). The mean age of participants was 23.4 (± 3.5) years. The female:male ratio of participants was 1.77:1 with 165 (64%) female and 93 (36%) male students. Of all students enrolled in the course “general pharmacology”, 68% were female and 32% male. In comparison, 65% of all medical students and 68% of first year medical students in Germany in 2013 were female [22]. A total of 316 out of 338 (94%) students participated in the final paper-based survey.

### Use of digital vs. non-digital media

**Fig 2:**
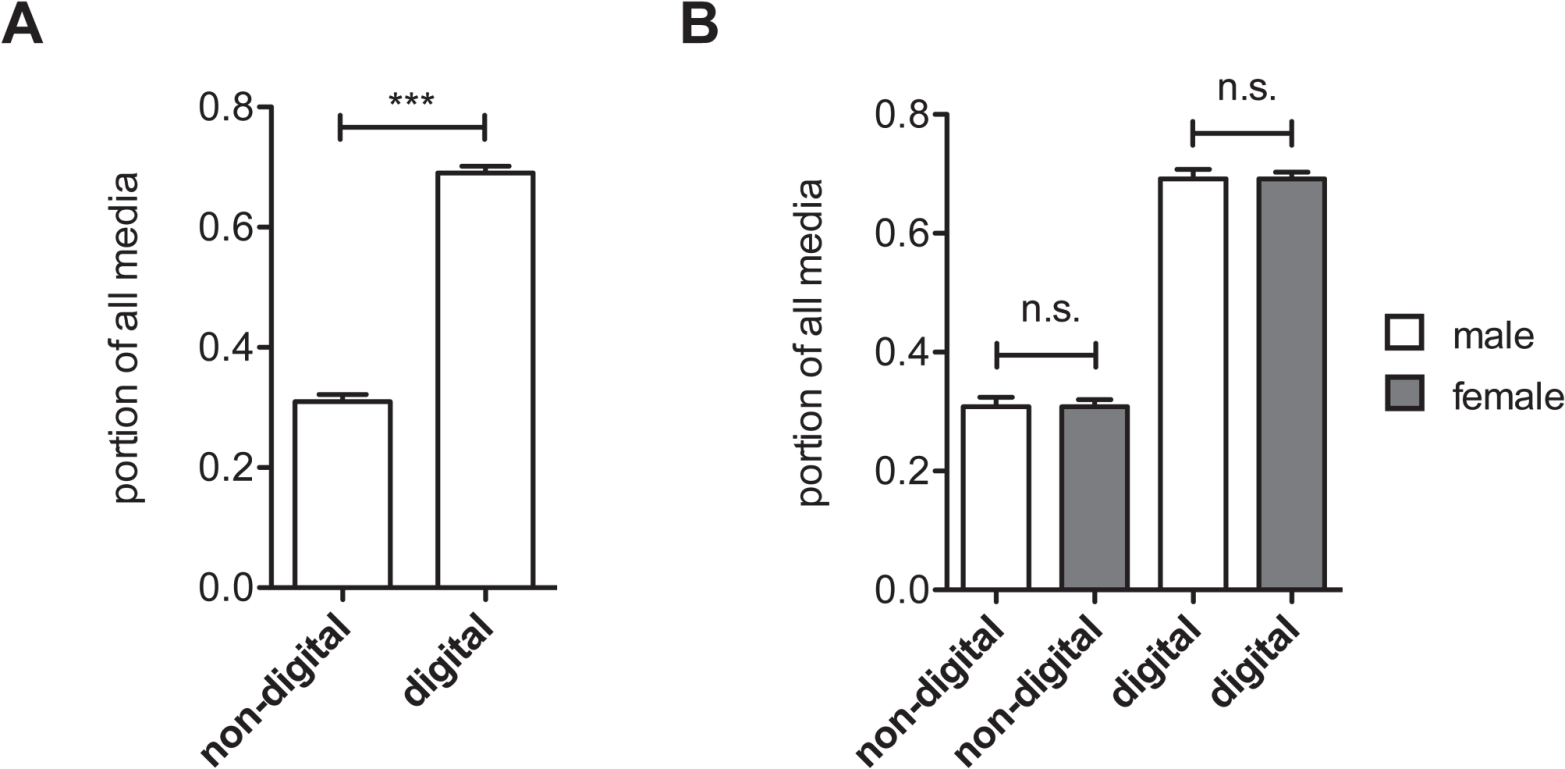
Use of digital and non-digital learning resources by students. A. Cumulative results of all study participants. B. Cumulative results of male and female students, respectively. Non-digital learning media included textbooks and personal notes. Digital learning media included apps, lecture slides, exam questions, internet search, podcasts, e-books and e-learning cases. n = 258, *** p < 0.01; n.s. = non-significant.

To gain insights into learning resource use by undergraduate medical students in pharmacology, we analyzed the overall employment of digital and non-digital learning media over the whole course period. Digital learning media were defined as electronic means of communication that deliver learning content via the internet and included smartphone applications (web-based or native apps), lecture slides (at TUM downloadable as Portable Document Format (PDF) files via the campus management system), online exam questions, internet search, podcasts, e-books and e-learning cases. Non-digital learning media were defined as paper-based resources and included printed textbooks (>300 or <300 pages, respectively) and personal notes by the students. All learning resources surveyed were available to students at TUM. Cumulative analysis of the 1^st^, 2^nd^ and 3^rd^ most used learning media revealed that study participants predominantly employed digital over non-digital learning resources (69 ± 7% vs. 31 ± 7%; p < 0.01; Fig 2A). Of note, no significant difference in the use of digital and non-digital media between male and female study participants was observed (Fig 2B).

### Quantitative ranking of learning media

**Fig 3:**
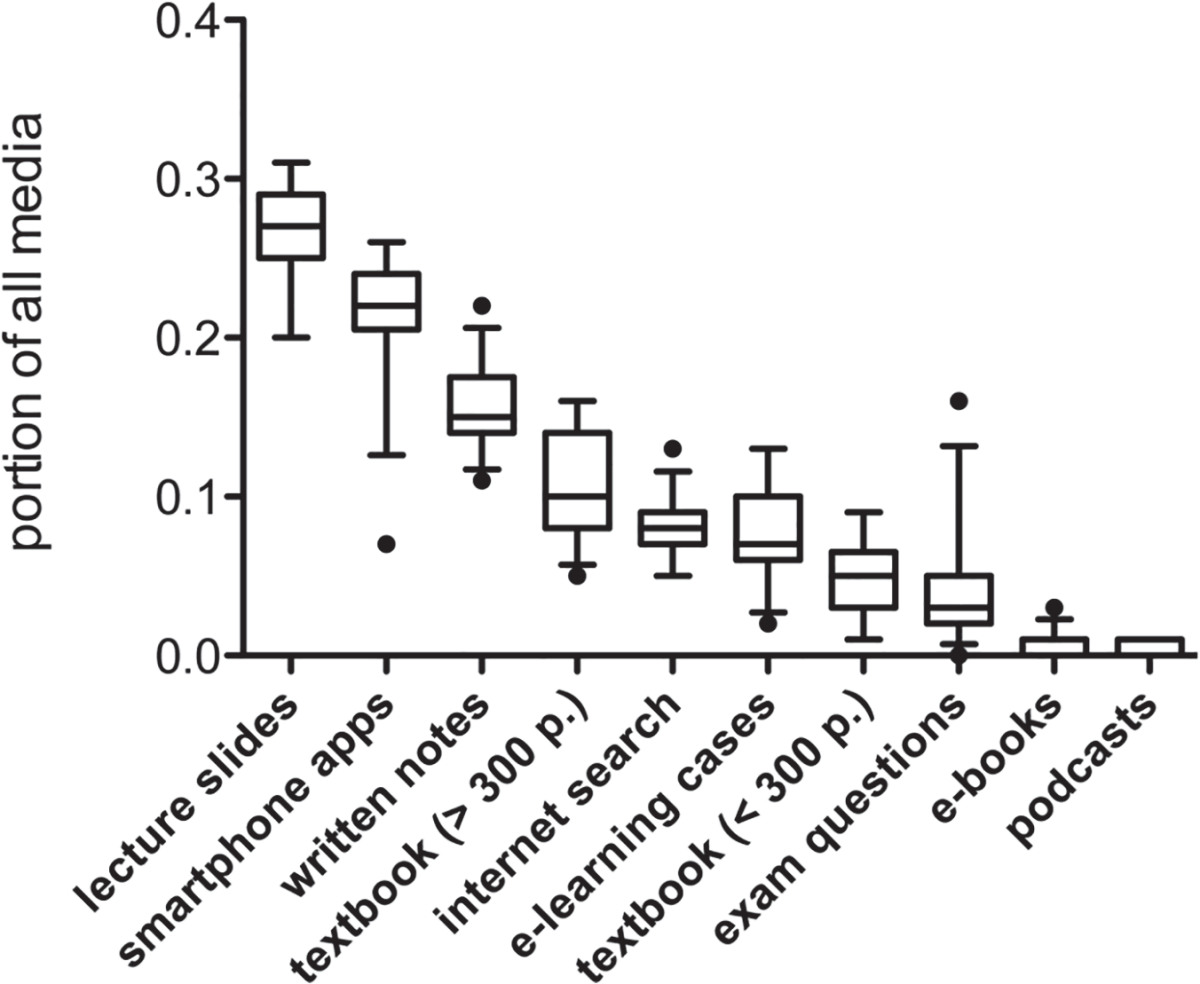
Quantitative ranking of learning resources during the course module. Box plots showing median, first and third quartile with whiskers representing the 5% and 95% percentile. Statistical outliers are shown as black dots. n = 258.

To delineate the quantitative significance of individual media for learning, we investigated the number of votes for each medium in relation to all responses. Fig 3 depicts the media ranking based on mean use in percent. The most utilized learning resources were lecture slides (26.8 ± 3.0%), apps (22.0 ± 3.7%) and personal notes (15.5 ± 2.7%), followed by textbooks > 300 pages (10.6 ± 3.3%), internet search (7.9 ± 1.6%) and e-learning cases (7.6 ± 3.0%). Other learning resources were only rarely used by the students, e.g. textbooks < 300 pages (4.8 ± 2.2%), e-books (0.7 ± 0.7%) or podcasts (0.3 ± 0.5%). Investigation of media use during the teaching period revealed similar results, while ranking of learning media differed slightly in the self-study period (S1 Fig).

Collectively, these data reveal a predominant use of digital learning resources by undergraduate medical students in pharmacology, and suggest that the relevance of individual learning resources varied between teaching and self-study periods of the same course module.

### Daily media use and comparison of teaching vs. self-study period

**Fig 4:**
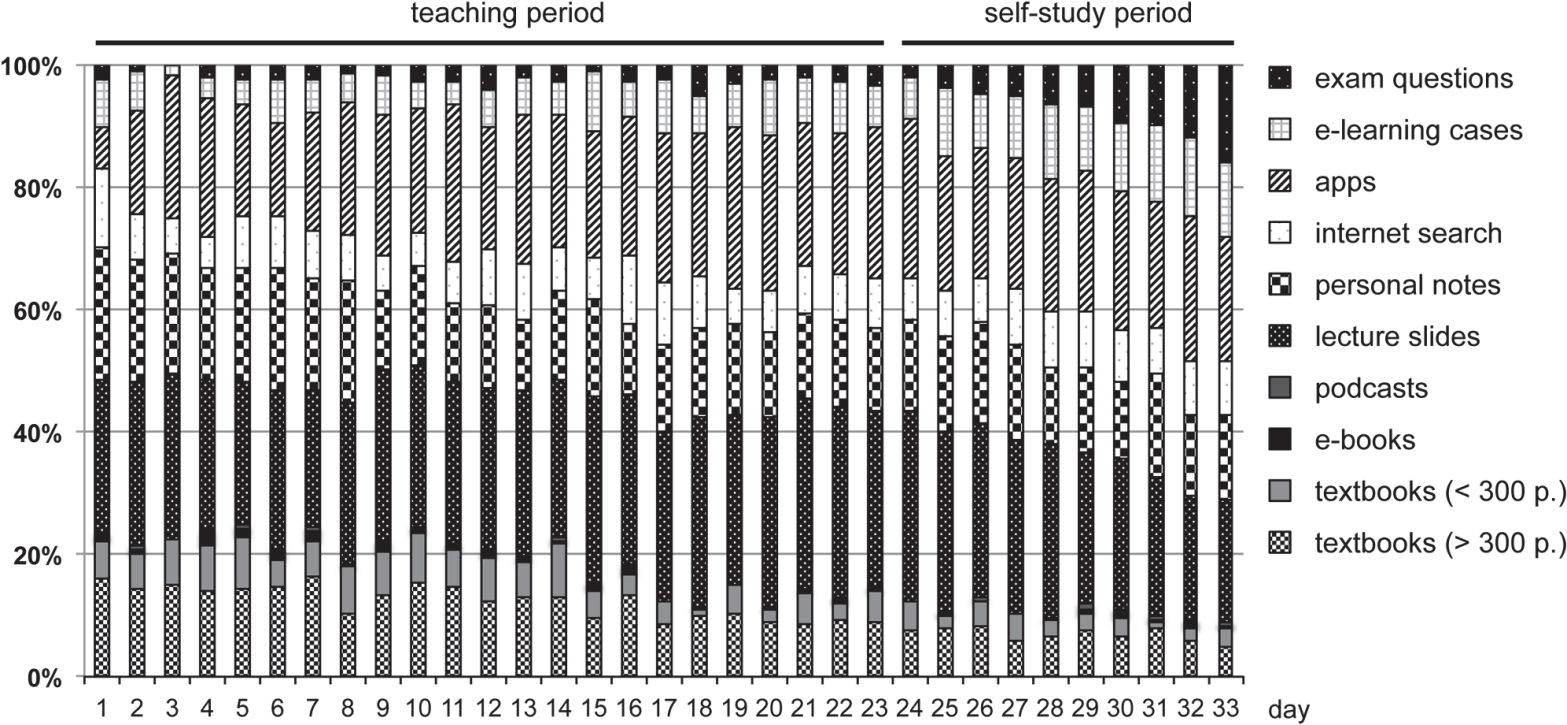
Analysis of daily media use. The stacked bars are depicting the ratio of each learning resources in relation to all learning media used per day in percent. Day 1–23 was teaching period, day 24–33 self-study period. The mean daily response rate in the online survey was 79.5 (± 18.2) students.

To monitor dynamic patterns of learning media use within the course module, we tracked the employment of individual media types at day-by-day resolution. As shown in Fig 4, the application of most learning media types was relatively constant, while some media exhibited noticeable differences during the teaching module. We performed comparative statistics to assess potential changes for individual learning resources between teaching (day 1–25) and self-study periods (day 26–35) (S2 Fig). Of the non-digital learning media, only textbooks (> 300 p) and textbooks (< 300 p.) were used significantly less in the self-study period when compared to the teaching period (-57%; P < 0.0001 and -53%; p < 0.01, respectively). Of the digital learning media, the use of exam questions (+176%; p < 0.01) and e-learning cases (+334%; p < 0.01) markedly increased in the self-study period. No significant changes were observed for other learning media.

### Qualitative analysis

To expand, cross-check and further understand data obtained from the quantitative analysis, semi-structured telephone interviews were conducted with a sub-cohort of survey participants. Representative statements are cited in Table 1. The interview participants endorsed central findings of the quantitative study, foremost the different usage patterns of textbooks, exam questions and e-learning cases in teaching vs. self-study periods. When asked for potential reasons for the decline of textbook use during the progression of the pharmacology course, a common theme that emerged from the interviews was the perceived benefit of textbooks for systematic knowledge acquisition of new topics, which is particularly required in the initial course phase. In contrast, exam questions and e-learning cases were seen as best suited for knowledge consolidation and transfer to clinical scenarios. Inquiring about the high but constant use of apps in both teaching and self-study periods, interview participants most often mentioned quick data access and concise presentation of information as the key advantages of these media. Finally, lecture slides by the educators were regarded as important framework for learning and guidance for (exam) relevant information.

**Table 1:** Qualitative results of semi-structured interviews (n = 11).

Theme	Code	Representative Statements
**Advantages of non-digital media**	Textbooks	*“I use books at the beginning [of the course] in order to get a detailed overview of the entire topic.”*
**Advantages of digital media**	Exam questions / E-learning cases	*“At the end [of the course] I like to learn with quizzes or [e-learning] cases because it is a playful repetition.”*
	Apps	*“The information [of the app] was very condensed*, *very useful for repetition.”*
	Apps	*“I liked apps because I could look up information quickly on the go.”*
	Lecture slides	*“I predominantly learned with the lecture slides because those contained all the relevant information I needed for the exam.”*

## Discussion

There is a paucity of information about the quantitative and qualitative use of learning resources by the current student generation in higher education. In this study we provide a comprehensive assessment of learning media use by undergraduate medical students in a genuine educational setting. Using a prospective mixed-method design with daily online surveys and final semi-structured interviews, we demonstrate a preponderance of digital resources for learning by both male and female students in pharmacology. We show that students employ a broad spectrum of learning media, of which lecture slides, apps and personal notes were most utilized, followed by textbooks (> 300 pages), internet search and e-learning cases. Finally, our results indicate that students`use of some learning resources is dynamic and varies between teaching and self-study periods.

### Students predominantly use digital learning resources

Our results revealed the predominant use of digital learning resources by 3^rd^ year undergraduate medical students enrolled in a pharmacology course in Germany. This finding is in accordance with studies conducted with US medical students on internal medicine clerkships that reported internet databases (e.g. Up-to-date) as a major information source [9–11]. In contrast, a focus group study with UK medical students after their first primary care attachment found non-digital media (notably textbooks) as the medium of choice for personal study [12]. This discrepancy may in part be explained by the heterogeneity of students with regard to the use and preference of digital learning sources and devices [16,23], or a non-representative study participants selection for which qualitative studies with smaller participant numbers (e.g. focus groups) are particularily prone [24].

An important finding of our study was that preference of digital and non-digital learning resources did not differ between male and female students. Imhof and co-workers assessed the computer use in a cohort of German university students and reported no gender differences, neither with regard to time spent on task nor with preferred activities at the computer [25]. These and our findings contrast earlier studies that reported significant higher computer and internet use by male students [26,27].

### Quantitative relevance of learning resources

Our survey data revealed the use of a broad spectrum of learning resources by undergraduate medical students in pharmacology. Textbooks, long regarded as the main medium for students`learning [28], accounted only for approx. 15% of most used learning resources in our study. This range is consistent with a recent study using self-reporting diaries in a cohort of UK medical students that found textbooks to account for approx. 25% of learning media [29], suggesting that the role of “traditional” learning resources in higher education is in a process of rapid transformation. Moreover, the efforts to adapt textbooks to the digital world, e.g. as e-books appears to be of limited success. In our study, we found e-books only rarely used by students despite the fact that most textbooks used at our institution are available in digital formats. These results underscore earlier findings by Woody et al. [13] who showed that the majority of students preferred printed over electronic textbooks irrespective of gender, computer use or technical affinity.

A surprising observation of our study was the importance of apps, ranking second of all learning media. Apps are applications optimized for mobile internet-enabled devices (smartphones or tablet-PCs), but are increasingly compatible to a broader range of computing devices (e.g. as web-based apps). We previously reported that approximately 80% of our students at TUM own a mobile internet-enabled device [30], which is in agreement with recent studies on smartphone ownership amongst college students in the UK [31,32]. It was reported that the majority of students have a positive attitude towards the use of these technologies for educational purposes [15,33]. However, as emphasized by Nguyen [17], the availability of high quality apps for learning and its curricular integration is still in its infancy. Moreover, several studies have reported a reluctance of many educators to recommend or use these new educational tools [8]. In pharmacology, a number of well-developed apps exist (e.g. drug repositories), which may explain the frequent use of apps in our study cohort. It is tempting to speculate that current undergraduate students are likely to embrace apps as a major medium for learning, if available in good quality and fitted to their curricular contents.

Finally, in spite of the broad use of novel learning media in our study cohort, our quantitative and qualitative analyses demonstrated a key role for learning media provided by educators (e.g. lecture slides or indirectly as written notes). While the role of the lecturer is likely to change in the future from a provider of information towards a facilitator of learning [34], our results further underscore that today`s students value educators`learning materials as means of guidance. Moreover, as institutions and educators increasingly make materials available online (e.g. via campus management systems or open-source learning platforms), there will be a reduced need to consult traditional learning sources, e.g. textbooks.

An interesting outcome of our study is the dynamic pattern of learning media use within a self-contained teaching module. When comparing teaching vs. pre-exam self-study periods, textbooks (> 300 p. and < 300 p.) were used significantly less (-57% and -53%, respectively) during self-study, while exam questions (+334%) and e-learning cases (+176%) were utilized more. A study by Briscoe et al. reported that both medical students and residents preferred printed over digital media for initial learning, however reasons behind their preferences remained unknown [35]. Our interview results suggest this observation may be due to the perceived benefit of textbooks for systematic knowledge acquisition of new topics.

### Limitations of this study

While this study adds to our understanding of learning media use in undergraduate medical education, there are limitations inherent to the methods applied in this study. First, both survey and interviews rely on self-report that may not be answered accurately or faithfully. Second, we obtained quantitative data on learning media use by an online questionnaire linked to an online learning platform used at TUM. This may have led to a selection bias for students with higher affinity for digital technologies. To estimate the potential impact of this bias, we conducted an additional paper-based survey with all course participants. The vast majority (96%) of students stated that they have regularly accessed the online platform during the course period, thus arguing against a predominant use by a non-representative sub-cohort.

Finally, our study cohort consisted of 338 undergraduate medical students enrolled in a basic pharmacology course at a single German medical faculty. While the demographic characteristics of our study cohort was representative for German medical schools, females were slightly underrepresented in the online survey when compared to all course participants (64% vs. 68%), which might have affected study outcomes. In addition, media use and usage patterns may differ for students of other disciplines, institutions or academic levels and is likely dependent on materials provided or recommended by the faculty. The present study is therefore exploratory in nature and serves as basis for future multi-center confirmatory studies with larger cohort sizes.

## Conclusion

To our knowledge, this is the first prospective cohort study of daily learning media use in pharmacology. Both quantitative and qualitative data revealed a high prevalence and acceptance of digital learning resources, in particular mobile applications. Our data suggest that “digital natives” are open to novel learning resources, however learning media provided by the educator remain a key source of information and guidance. Further studies are needed to investigate what kind of learning media (or combination thereof) are associated with the best learning outcome in the current cohort of undergraduate students. In addition, further work needs to be done to examine cross-country differences in availability, use or development of digital resources in higher education. This will likely have important implications for the implementation of new learning media and technologies in academic programs and educational institutions. These research questions will become more complex with the fast pace of technological change and the progression of today`s students to the educators of tomorrow.

## Supporting Information

S1 Fig Quantitative ranking of learning resources in different course periods. A. Teaching period. B. Self-study period. Box plots showing median, first and third quartile with whiskers representing the 5% and 95% percentile. Statistical outliers are shown as black dots. n = 258.
(TIF)

S2 Fig Comparison of learning media use in teaching vs. self-study periods. Dot plot charts of most used learning media per day. Each data point represents the mean cumulative responses for a learning medium of a single day in relation to all media in percent. The mean daily participation rate in the online survey was 79.5 (± 18.2).
(TIF)

S1 Table Daily response rate of online survey. (DOCX)
