# Supplementary figures and images for: Use of Learning Media by Undergraduate Medical Students in Pharmacology: A Prospective Cohort Study
Source: PLoS One. 2015 Apr 7;10(4):e0122624. doi: 10.1371/journal.pone.0122624 (PMC4388621; doi:10.1371/journal.pone.0122624)

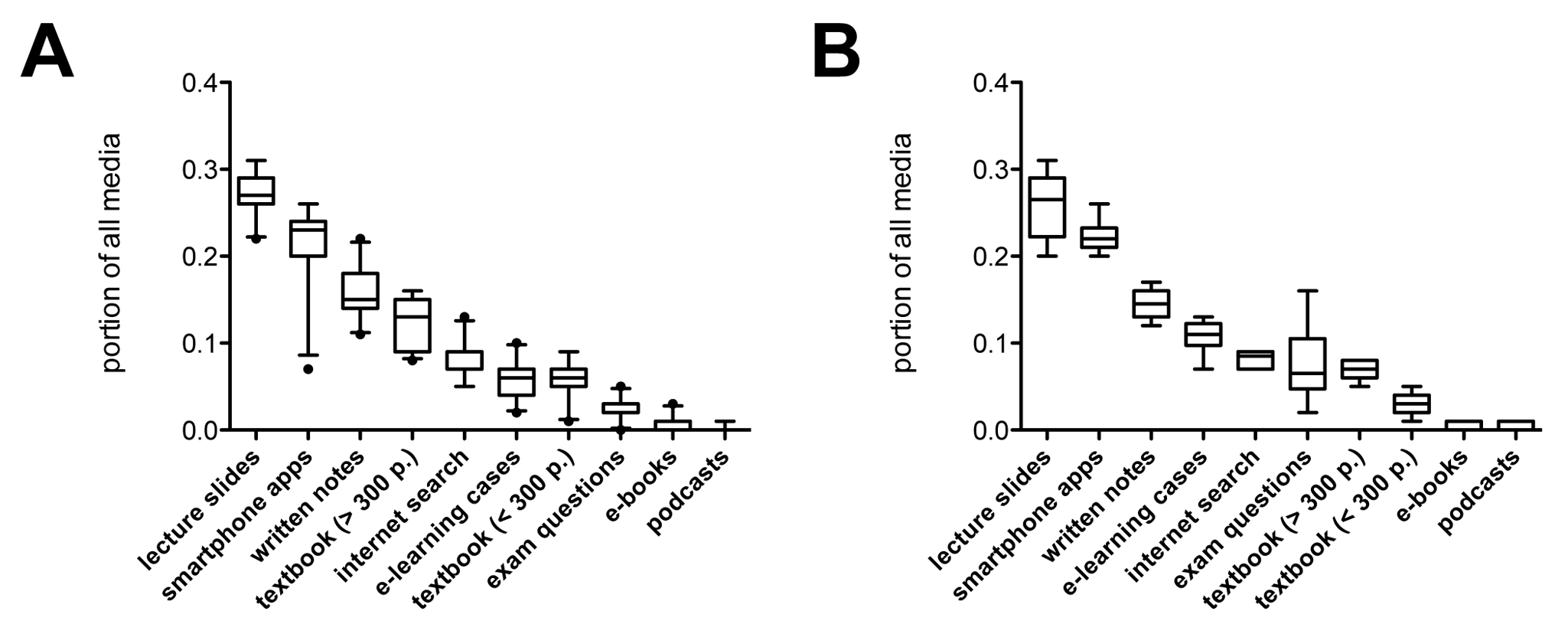

Supplement: S1 Fig — A. Teaching period. B. Self-study period. Box plots showing median, first and third quartile with whiskers representing the 5% and 95% percentile. Statistical outliers are shown as black dots. n = 258. (TIF) [file pone.0122624.s001.tif]

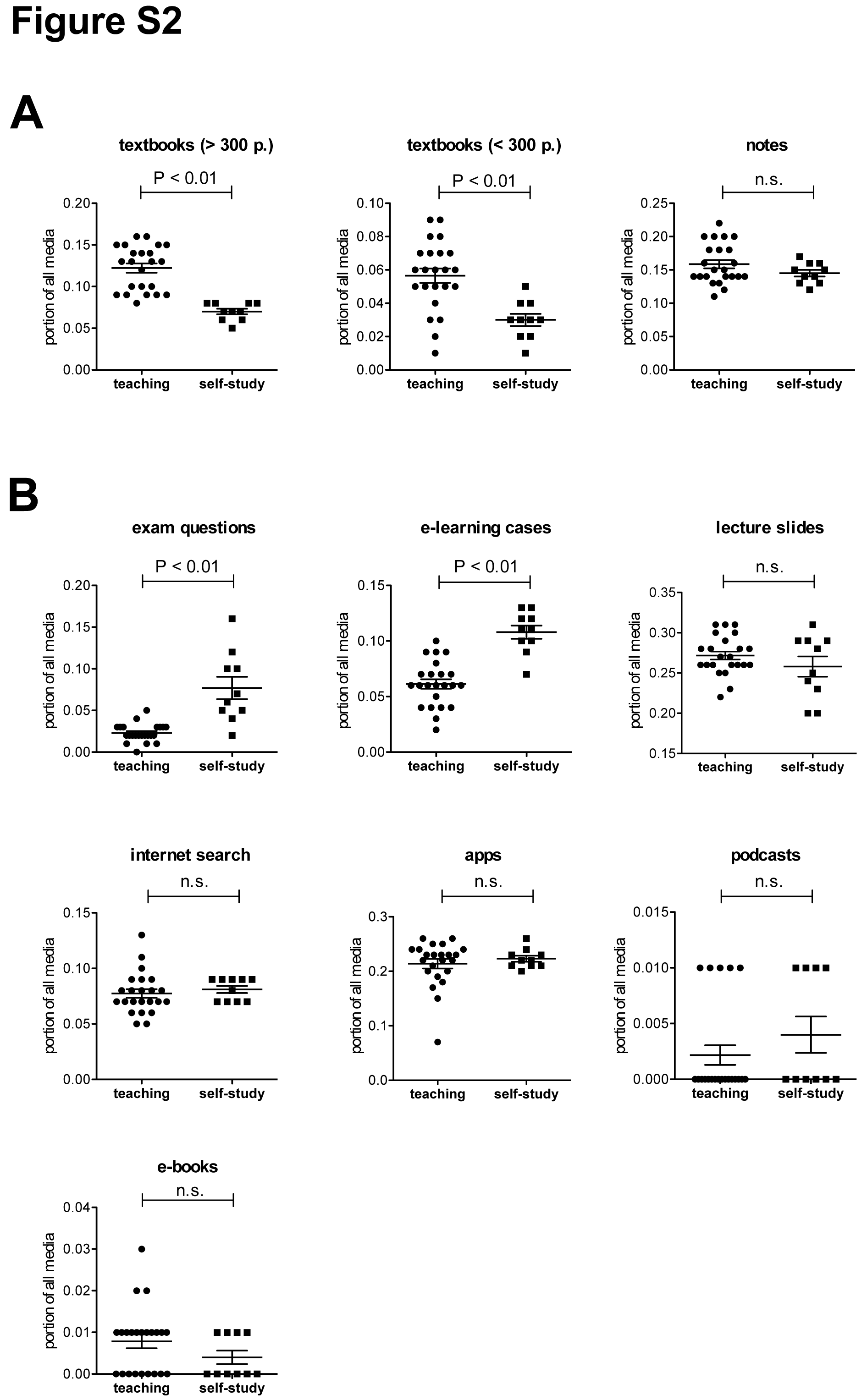

Supplement: S2 Fig — Dot plot charts of most used learning media per day. Each data point represents the mean cumulative responses for a learning medium of a single day in relation to all media in percent. The mean daily participation rate in the online survey was 79.5 (± 18.2). (TIF) [file pone.0122624.s002.tif]
