# Supplementary material for: Use of Learning Media by Undergraduate Medical Students in Pharmacology: A Prospective Cohort Study
Source: PLoS One. 2015 Apr 7;10(4):e0122624. doi: 10.1371/journal.pone.0122624 (PMC4388621; doi:10.1371/journal.pone.0122624)
Supplement: S1 Table — (DOCX) [file pone.0122624.s003.docx]

| **Course Period** | **Day** | **Participant number** |
| --- | --- | --- |
|  |  |  |
| teaching | 1* | 29 |
| teaching | 2 | 75 |
| teaching | 3 | 68 |
| teaching | 4 | 56 |
| teaching | 5 | 50 |
| teaching | 6 | 74 |
| teaching | 7 | 59 |
| teaching | 8 | 69 |
| teaching | 9 | 67 |
| teaching | 10 | 57 |
| teaching | 11 | 61 |
| teaching | 12 | 61 |
| teaching | 13 | 75 |
| teaching | 14 | 69 |
| teaching | 15 | 77 |
| teaching | 16 | 73 |
| teaching | 17 | 62 |
| teaching | 18 | 59 |
| teaching | 19 | 74 |
| teaching | 20 | 84 |
| teaching | 21 | 85 |
| teaching | 22 | 82 |
| teaching | 23 | 97 |
| teaching | 24 | 79 |
| teaching | 25 | 79 |
| self-study | 26 | 91 |
| self-study | 27 | 89 |
| self-study | 28 | 115 |
| self-study | 29 | 105 |
| self-study | 30 | 108 |
| self-study | 31 | 105 |
| self-study | 32 | 110 |
| self-study | 33 | 109 |
| self-study | 34 | 100 |
| self-study | 35* | 24 |
|  |  |  |
|  | Mean | 79.5 |
|  | SD | 18.2 |
|  | SEM | 3.2 |

**Table S1. Daily response rate of online survey.**

Responses of day 1 and 35 were omitted due to low participation numbers.
